# Distinct Responses of Biofilm Carbon Metabolism to Nanoplastics with Different Surface Modifications

**DOI:** 10.3390/ijerph19159148

**Published:** 2022-07-27

**Authors:** Yang Liu, Weiyu Li, Chunmei Tao, Junjie Zhao, Hongmei Zhang, Lingzhan Miao, Yong Pang, Jun Hou

**Affiliations:** 1 College of Hydrology and Water Resources, Hohai University, Nanjing 210098, China; lioyang203@126.com (Y.L.); ypang@hhu.edu.cn (Y.P.); 2 Jiangsu Environmental Engineering Technology Co., Ltd., Nanjing 210036, China; 3 Key Laboratory of Integrated Regulation and Resources Development on Shallow Lakes, Ministry of Education, College of Environment, Hohai University, Nanjing 210098, China; 2014050116@hhu.edu.cn (W.L.); lzmiao@hhu.edu.cn (L.M.); hjy_hj@hhu.edu.cn (J.H.); 4 Lianyungang Water Conservancy Bureau, Lianyungang 222006, China; tcm13851283076@163.com; 5 Key Laboratory of Waterway Traffic Environmental Protection Technology, Tianjin Research Institute for Water Transport Engineering, M.O.T, Tianjin 300456, China; 6 Shannan Ecological Environment Monitoring Center, Tibet 856100, China

**Keywords:** nanoplastics, biofilm, toxic effects, carbon metabolism

## Abstract

Recently, there is an increasing concern regarding the toxicity of nanoplastics (NPs) on freshwater organisms. However, knowledge about the potential impacts of NPs with different surface modification on freshwater biofilms is still very limited. In this research, biofilms were cultured in lab and exposed to nano polystyrene (PS) beads: non-functionalized PS NPs, PS-COOH NPs, and the carbon source utilization of biofilms were measured by BIOLOG ECO microplates. The results showed that both two types of PS NPs significantly reduced the total carbon metabolic activity of biofilms, compared with the controls, whereas the carbon metabolic rate increased notably, especially for the PS-COOH NPs treatments at day 14. Moreover, results from six categories of carbon sources analysis suggested that PS NPs with different surface chemical properties exhibit distinct effects on the carbon utilization of biofilms, and the divergent changes of the specific carbon source category were observed at day 21 from the two PS NPs treatments. In addition, the metabolic functional diversity of biofilms were not altered by the PS NPs treatments. These findings highlighted that chemical properties of NPs play an important role in the toxic effects on the carbon metabolism activities of the biofilms. This study offers new insights that nanoplastics of different chemical characteristics have the ability to affect the microbial-mediated carbon cycling process in aquatic ecosystems.

## 1. Introduction

With more nanoplastics (NPs) entering into freshwater systems, the potential impacts of NPs have drawn increasing concerns in the past decade due to their potential hazardous properties on aquatic organisms [1,2]. In addition, NPs can be formed during the fragmentation processes of MPs in an aquatic environment, leading to the concentrations of NPs to surge with time [3,4]. Previous studies have reported that NPs show negative toxicity to cells by permeating into biological barriers and lipid membranes [5] and then damage the membranes structure and nutrients exchange within the cell systems [6,7]. As a result, the exposure to NPs resulted in significant changes in the molecule diffusion and even the gene expression of aquatic organisms [6,8]. Therefore, the potential impacts of NPs in freshwater environments should be paid more attention.

During their transport in freshwater, NPs would come into contact with aquatic life, such as periphytic biofilms [9,10]. As ubiquitous in aquatic environments, biofilms are microbial aggregates of multi-species, mainly including algae and bacteria [11]. Biofilms have been considered as an essential environmental medium, providing primary productivity that support higher trophic levels and participating in the biogeochemical cycle in aquatic environments [11]. Furthermore, biofilms can serve as a bioindicator for evaluating the pollutant effects on aquatic ecosystems [12,13]. Therefore, the assessment of microbial functional characteristics related to the biogeochemical cycles of biofilms is very important to understand their response to external pollutants, for example NPs in aquatic ecosystems. Recent studies have documented the effects of nanoplastics on algae and microbes in aquatic ecosystems. For example, polystyrene (PS) NPs (50 nm) increased the production of reactive oxygen species (ROS) and had a negative impact on the inorganic nitrogen conversion efficiency of marine bacterium alkalophilic Halomonas [14]. In addition, Lu et al. found that a low concentration (10 mg/L) of 100 nm plastic particles inhibited the reproduction of Chlorella pyrenoidosa, while a high-concentration (100 mg/L) exposure significantly increased the reproduction rate [15]. Previously, Guo et al. studied the effect of nano-PS beads with different concentrations (1, 5 and 10 mg/L) on freshwater biofilm microbial metabolic functions [16], and Miao et al. studied the acute effects of nano-PS beads with different particle sizes and concentrations on the functional enzymes of periphytic biofilms [10]. However, there is limited information about the effects of nanoplastics with different surface modifications on the microbial functions of freshwater biofilms. After entering into the water, plastics could be affected by a variety of environmental factors, resulting in significant changes in their surface characteristics and forming PS NPs and PS-COOH NPs. Due to different surface functional groups, nano-plastics showed different toxic effects on aquatic microorganisms [10,14].

Therefore, in this study, the responses of biofilm to nanoplastics with different surface modification were investigated focusing on the carbon metabolism function of biofilms. Freshwater biofilms were incubated in lab and then exposed to different surface-modified PS NPs (100 nm): non-functionalized PS NPs, PS-COOH NPs. The dynamic responses of biofilm carbon metabolism were measured at the 7th, 14th, and 21st day respectively, by using BIOLOG ECO microplates. The purpose of this study is to explore the toxic effects of different surface-modified nano-plastics on freshwater epiphytic biofilm and their related carbon biogeochemical cycles.

## 2. Materials and Methods

### 2.1. Nano-Plastics Preparation and Characterization

Commercially available PS pellets (100 nm) were obtained from BaseLine Chromtech Research Centre (Tianjin, China). The polystyrene pellets were monodisperse and supplied in 10 mL aqueous suspensions. Two kinds of PS pellets were used in the exposure experiments: non-functionalized PS and carboxyl PS (PS-COOH). During the transport of NPs in freshwater, their surface characteristics may be altered by the various environmental factors and previous studies have demonstrated that NPs with different surface modifications exhibit different toxic effects on microorganisms [10,14]. Therefore, these two types of NPs were chosen to determine the effects of different surface chemical properties on epiphytic biofilms. Prepared stock suspensions of PS (100 mg/L) underwent an ultrasound (20 °C, 250 W, 40 kHz) for 10 min [10] and then dialyzed to remove the sodium dodecyl sulfate (SDS; surfactant to prevent particle aggregation and matain particle size) present within the PS solution [17] before the PS-NPs exposure experiments. In this study, a Zetasizer Nano ZSP instrument (Malvern Instruments, Malvern, UK) was used to measure the hydrodynamic diameter and zeta potential of PS NPs. The surface morphology of PS particles was observed by scanning electron microscope (SEM) [10].

### 2.2. Biofilm Cultivation

Biofilm samples were scraped from the benthic rocks of Xuanwu Lake in Nanjing, Eastern China (118.7837° E, 32.0692° N) with sterile brushes. Large granular particles and micro animals attached to pebbles were removed, and then the biofilm was stored in a sterile tank and brought back to the laboratory with ice within 1 h. Meanwhile, overlying water from Xuanwu lake was also obtained to determine water quality parameters, which were finally determined as: pH = 7.7; total nitrogen (TN) = 2.3 mg/L; total phosphorus (TP) = 0.13 mg/L; ${\mathrm{NH}}_{4}^{+}$-N = 0.62 mg/L; and ${\mathrm{NO}}_{3}^{-}$-N = 0.85 mg/L.

The biofilm samples were transferred to a laboratory and cultivated in an indoor flume in a greenhouse described in our previous study [18] to simulate natural conditions. The biofilms were developed on pebbles and WC medium (Table S1) were added weekly to maintain the nutrition [19]. After 6 weeks of cultivation, the mature biofilms were used to further experiments.

### 2.3. Exposure Experiment

After cultivation, biofilms on pebbles were transferred to Erlenmeyer flasks, containing 120 mL of water and 10 pebbles. The biofilms were firstly acclimated for 2 h [20], and then the NPs exposure experiments started. Two types of PS NPs were used, including non-functionalized, un-functionalized PS (100 nm), carboxyl PS (100 nm, PS-COOH), and the exposure concentration were set at 10 mg/L. These coatings simulated the variety of engineered and naturally occurring surface modifications of plastics [21,22], enabling us to determine how surface chemistry influences the effect on freshwater biofilms. The flasks without adding any PS NPs served as controls. The exposure treatments and the control were replicated three times, and the exposure experiment lasted for 21 days [10]. During the exposure process, biofilm samples were collected and measured on the 7th, 14th, and 21st day respectively.

### 2.4. Community-Level Physiological Profiling

#### 2.4.1. BIOLOG ECO Microplate Cultivation

Average well color development (AWCD) was measured using BIOLOG ECO microplates (Hayward, CA, USA) (see Supplementary Material SI), which can be used to assess the ability of microbes to metabolize carbon sources. The details were given in our previous study [16]. Briefly, the biofilm samples were suspended and ultrasonicated. Then, the solution was gradually diluted until an optical density (OD) value reached 0.05 ${\mathrm{cm}}^{-1}$ at 590 nm. Next, the corresponding diluted suspension (150 μL) was added into the wells (150 μL) of the BIOLOG EcoPlate, which was then placed into an incubator at 25 °C for 6 days under shading [23]. Besides, The absorbance values at 590 nm wavelength were read at certain time intervals for 6 days using a multifunctional enzyme label tester [16].

#### 2.4.2. Determination of Average Well Color Development Values

In order to determine the microbes’ ability to utilize various carbon sources and characterize their metabolic proficiency, some corresponding indicators, such as the average well-color development (AWCD) and three metabolic functional diversity indices: Shannon–Wiener diversity index (H′), Simpson diversity index (D) and Shannon evenness index (E) were measured according to the following formula:

(1) $$\;\mathrm{AWCD}=\overset{n}{\underset{i=1}{\sum }}\left(C_{i}-R\right)/n$$

where C_i_ refers to OD 590 of the absorbance value of each well, R is the OD value of the control well, and n is the amount of reactive wells [24,25]. In addition, (C_i_ − R) values less than 0.06 were counted as zero [24].

The non-linear fitting curve of AWCD varying with time was fitted by the logistic growth equation (Equation (2)).

(2) $$\mathrm{AWCD}=\frac{K}{1+e^{-p\left(t-s\right)}}$$

where K denotes the maximum value of the AWCD and utilization capacity of carbon sources; p represents maximum exponential rate of AWCD change, which indicates the highest carbon metabolism rate; s illustrates the time at which AWCD is the half carrying capacity value (K/2); and t is an independent variable called incubation time.

By plotting the sigmoidal color development curves of the corrected AWCD of each biofilm versus its read time, each plate’s overall metabolic activity can be read clearly. The greater the degree of sample variation, the higher the carbon utilization capacity. The absolute value of the development curve slope was measured as the metabolic rate [25].

Furthermore, based on the determination of BIOLOG ECO microplates, the metabolic functional diversity indices of biofilms were calculated, including Shannon–Wiener diversity index (H′) and Simpson diversity index (D). These metabolic functional diversity indices can reflect the metabolic diversity of microbial communities and are widely adopted in previous studies [25,26]. The calculation formula were provided in the supplementary material.

### 2.5. Statistical Analysis

All assays were conducted in three replicates, and data were expressed as the mean ± standard deviation. Significant differences between treatments were analyzed in Origin 2022 using one-way ANOVA followed by post hoc Tukey test; significance level was set as 0.05.

## 3. Results

### 3.1. Characterization of PS Nanoplastics

In this study, the morphology of PS NPs were observed to be spherical from the SEM images (Figure S1). The particle size distribution of PS NPs in Milli-Q water and experimental solution were measured [10], and the results are shown in Table 1. Obvious aggregation of PS NPs for both non-functionalized PS and carboxyl PS were observed in the experimental solutions with much higher hydrodynamic diameters than those in Milli-Q water (*p* < 0.05). Moreover, lower absolute values of zeta potential of both non-functionalized PS and carboxyl PS were observed in experimental solution than those in Milli-Q water (*p* < 0.05), indicating that the electrostatic repulsions between nano particles were decreased [10,14]. Accordingly, the decreased surface charge of PS NPs could be explained by the screening effect due to the high ion strength [27], leading to aggregation of PS NPs.

### 3.2. Effects on the Biofilms

#### 3.2.1. Effects on the Total Carbon Metabolism of Biofilms

**Figure 1:**
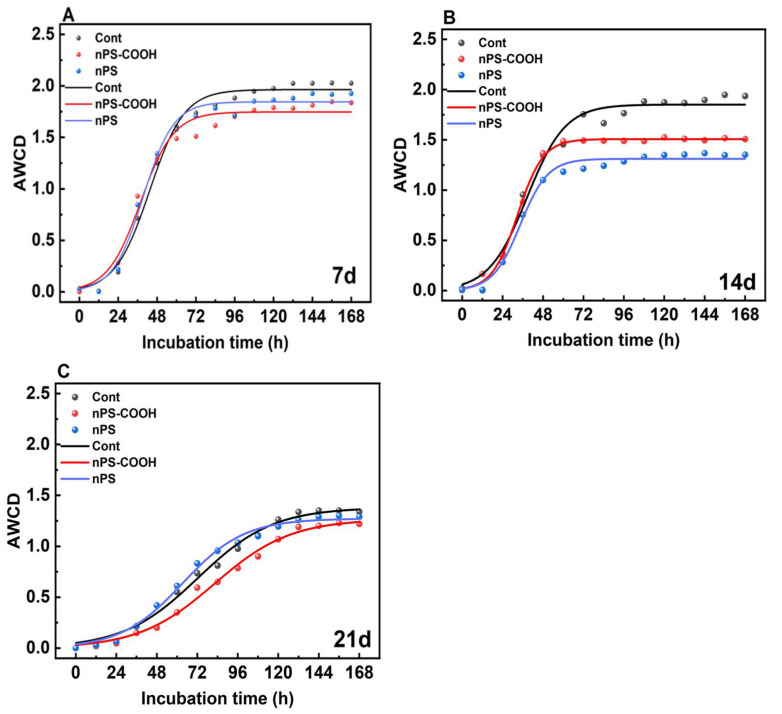
Comparison of carbon metabolic capacity of biofilms exposed to PS NPs with different surface modifications (nPS and nPS-COOH) at day 7 (**A**), day 14 (**B**), and day 21 (**C**) (*n* = 3).

Related to carbon source utilization, the AWCD can represent the overall metabolic activity of biofilm communities [24]. The AWCD values of biofilm communities measured at different exposure times were shown in Figure 1. The dynamic increase of the AWCD exhibited sigmoid patterns with increasing time, and the nonlinear fitting curve shown in Equation (1) was used to fit the curve. Specifically, the AWCD of all samples exhibited a lag phase for the initial 24 h, suggesting that the consumption of carbon source remained at a low level. After that, the activity of biofilm communities increased significantly, indicating that the metabolic process of all microplates entered an efficient stage. The activity increased steadily after 80 h. After about 120 h of incubation, the metabolic utilization capacity was stable, and AWCD remained at a stable value (K).

There was significant difference between the AWCD values of the experimental groups exposed to nanoplastics and the control group. After reaching the peak value and stabilizing at different times, then, the total carbon metabolism capacity (K) of biofilms was determined by fitting by Equation (2) at the stable stage.

**Figure 2:**
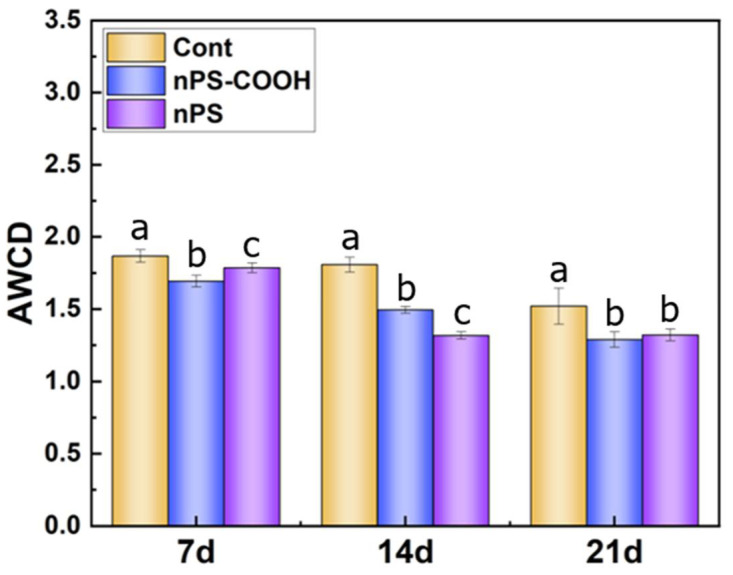
Total carbon metabolism capacity of biofilms (fitted by Equation (2) at the stable stage) exposed to PS NPs with different surface modifications at day 7, day 14, and day 21 (*n* = 3). Different letters represent significant differences among the PS treatments (*p* < 0.05).

The K from the logistic growth equation represents the maximum value of the AWCD and utilization capacity of carbon sources, and the K values of biofilm communities were shown in Figure 2. PS NPs treatments significantly changed the biofilm carbon metabolism during the whole dynamic monitoring. The AWCD were obviously lower in both non-functionalized PS and carboxyl PS exposure treatments, compared with the control tests (*p* < 0.05). Specifically, at day 7, biofilms from the nPS-COOH treatments exhibited a value of AWCD with 1.79 ± 0.041, significantly lower than those from non-functionalized PS treatment and the controls. At day 14, biofilms from the non-functionalized PS treatments exhibited lowest AWCD. After 21 days of exposure, biofilms from the non-functionalized PS and carboxyl PS treatments exhibited a similar AWCD, which was significantly lower than those from the controls. These results showed that the PS NPs could significantly reduce the total carbon metabolic activity of biofilms, and the surface modification of PS NPs could lead to distinct effects of NPs on biofilms.

Furthermore, the total AWCD is a result of averaging the metabolic activity of 31 carbon sources, and thus, although the total metabolic activity did not significantly change, it does not totally mean that there are no changes of the specific metabolic activity of biofilm communities. Therefore, more detailed effects of NPs on the specific types of carbon source metabolism of biofilms should be explored further.

#### 3.2.2. Effects on the Carbon Metabolism Rate of Biofilm

**Figure 3:**
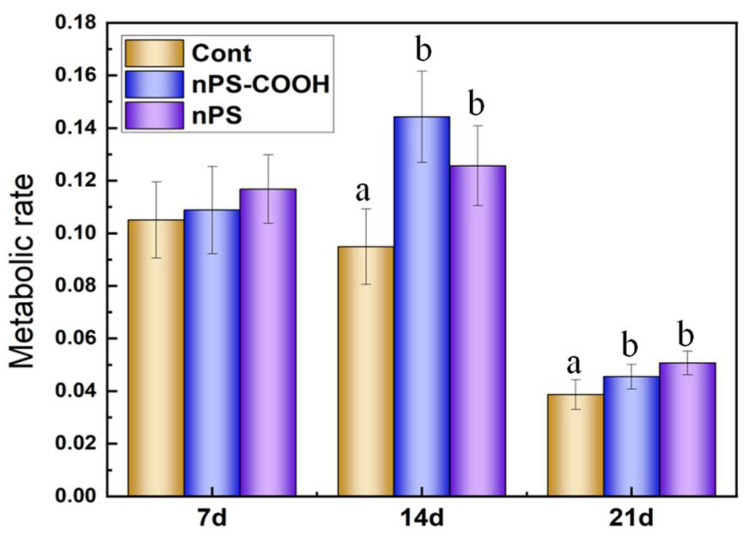
Comparison of carbon metabolic rate of biofilms (fitted by Equation (2) at the stable stage) exposed to PS NPs with different surface modifications at day 7, day 14, and day 21 (*n* = 3). Different letters represent significant differences among the PS treatments (*p* < 0.05).

The slope of each AWCD curve (labeled p) represents the maximum rate of metabolic activity. The *p* values of biofilm communities were shown in Figure 3. On the whole, similar levels of the carbon metabolic rate were observed on the 7th day and 14th day, while obvious declines were observed on the 21st day, likely due to the changes of microbial communities in biofilms with the exposure time increasing. As for the different treatments, after PS NPs treatments, the carbon metabolic rate of the exposure groups exhibited different changing patterns. Specifically, on the 7th day, the rate value of three treatments was at a similar level, indicating that NPs exposure does not affect the carbon metabolic efficiency of microorganisms at the early stage. While, on the 14th and 21st day, the rate values of nPS treatments were lower than that of controls, indicating that the carbon metabolic efficiency of microorganisms was promoted by NPs exposure at this stage.

#### 3.2.3. Effects on AWCD of Biochemical Categories

**Figure 4:**
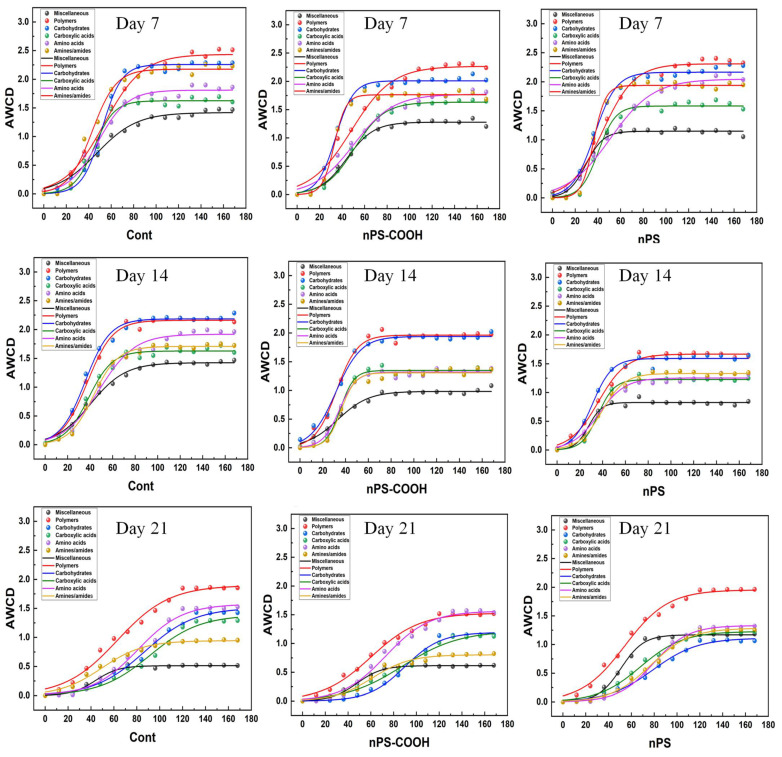
Comparison of metabolic capacity of the six biochemical categories of carbon of biofilms exposed to PS NPs with different surface modifications at day 7, day 14, and day 21 (*n* = 3).

According to the previous studies, the 31 carbon sources from the microplates were classified into six biochemical categories, including miscellaneous, polymers, carbohydrates, carboxyllic acids, amino acids, and amines/amides to assess metabolic functions in a physiologically relevant approach [16]. In general, the utilization activity for these six carbon sources of biofilms showed an increasing trend over time (Figure 4). At the first 12 h, the AWCD values of six types of carbon sources in each group remained relatively low, indicating that the metabolic consumption level of biofilms was low and the microbial activity was not strong at this stage. After 12 h, the AWCD values of each group began to increase significantly and the microbial activity increased. In addition, the utilization degrees of various carbon sources in each experimental group reached the maximum at different times and tended to be stable.

At day 7, the NPs exposure did not significantly affect the metabolic capacity of the six biochemical categories. While, at day 14, the metabolic capacity of the six carbon categories of biofilms was generally decreased, especially for the miscellaneous, carboxyllic acids, amino acids, and amines/amides. Interestingly, the divergent changes of the specific carbon source categories were observed at day 21 from the two PS NPs treatments. As for the PS-COOH NPs, the metabolic capacity of polymers, carbohydrates were significantly inhibited. While, in the non-functionalized PS NPs treatments, the metabolic capacity of polymers was not altered, meanwhile, the metabolic capacity of carbohydrates was inhibited. Moreover, the metabolic capacity of miscellaneous and amines/amides was obviously increased by the exposure to PS NPs. Therefore, it can be concluded that nano-plastics show obviously toxic effects on the metabolic capacity of biofilm on different carbon sources, and the dynamic responses of the specific carbon source categories of biofilms to PS NPs with various surface modifications are significantly different.

**Figure 5:**
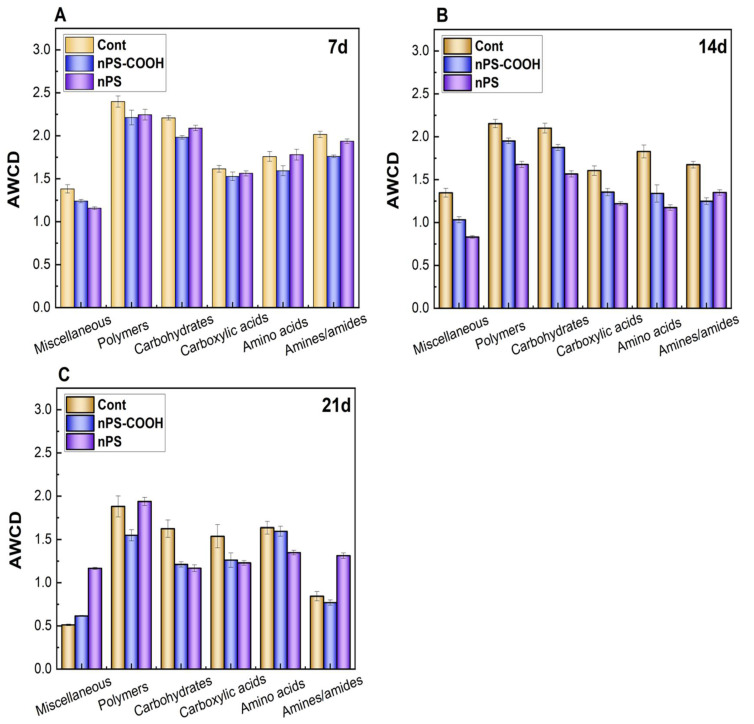
The metabolism capacity of the six biochemical categories carbon of biofilms (at the stable stage) exposed to PS NPs with different surface modifications at day 7 (**A**), day 14 (**B**), and day 21 (**C**) (*n* = 3).

The variation in AWCD of six types of carbon sources at different time points was displayed in Figure 5. The results presented here indicated that biofilms exhibit various metabolic capacities of the six biochemical categories carbon and the exposure to different surface modifications of PS NPs lead to different responses of biofilms. In detail, all biofilm samples showed preference for the carbohydrates and polymers, which were consistent with previous studies [28,29]. After NP exposure, miscellaneous and amines were utilized the least among the six categories of carbon sources. Moreover, the metabolic capacity of the six biochemical categories of carbon exhibited differences on the 7th and 14th days (*p* < 0.05), with no significant differences on the 21st day. It shows that PS beads with different surface characteristics have different degrees of inhibition on the ability of biofilm microorganisms to consume various carbon sources and have a certain impact on the preference of microorganisms for different carbon sources, but the trend is not significant with time passing.

#### 3.2.4. Effect on the Functional Diversity Indices

**Figure 6:**
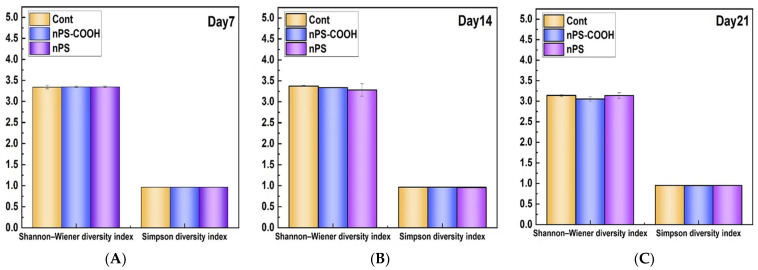
Comparison of metabolic functional diversity indices of biofilms exposed to PS NPs with different surface modifications at day 7 (**A**), day 14 (**B**), and day 21 (**C**) (n = 3).

The Shannon–Wiener diversity index ($H^{\prime }$) and Simpson diversity index (D) were calculated to represent the microbial metabolic functional diversity, reflecting the carbon source utilization heterogeneity and community structures of biofilms to a certain degree [26]. Accordingly, these two indices are linearly related to the relative abundance of the different carbon sources (evenness) and the $\log_{2}$ of the number of utilized carbon sources (richness). As displayed in Figure 6, the exposure of PS NPs did not lead to significant differences of the Shannon and Simpson index for both non-functionalized PS and carboxyl PS treatments. These results suggested that the metabolic functional diversity of biofilms was not altered by the PS NPs treatments, while the carbon metabolic capacity of biofilms was significantly changed.

## 4. Discussion

### 4.1. Responses to Different Surface Modifications of Polystyrene Nanoplastics

The NPs entering into freshwater environment may interact with microbial community and then accumulate in benthic habitat, which may affect the community structure and metabolic activity of biofilms. Previously, the surface modification has been demonstrated to influence the toxic effects of PS NPs on aquatic organisms [14,30]. Also, a previous study reported the effect of plastic surface characteristics (control, PS NPs, nPS-COOH NPs and nPS-${\mathrm{NH}}_{2}$ NPs) on the activity of biofilm three functional enzymes, including β-glucosidase (GLU), leucine aminopeptidase (LAP), and alkaline phosphatase (AKP) [10]. Their research results showed that different toxic effects of PS NPs on the three functional enzymes are observed dependent on the surface modification, and positively charged PS NPs (amide-modified) exhibited the highest toxicity to biofilms, especially on the activities of GLU and LAP. The exposure to PS NPs, nPS-COOH NPs, and nPS-${\mathrm{NH}}_{2}$ NPs led to similar inhibition of the activity of AKP within the experimental period. Also, a previous study reported the effects of PS NPs with different concentrations (1, 5, and 10 mg/L) on biofilm carbon metabolism [16]. It is found that the rate and activity of carbon metabolism of biofilm varied with PS concentration and that the functional diversity indices did not change significantly. In specific, nano-sized polystyrene at 1 mg/L and 5 mg/L, concentrations did not show strong disturbance on the freshwater microbial community, while the 10 mg/L PS concentration group significantly inhibited biofilm carbon metabolism compared to the control group (*p* < 0.05). In this study, the same concentrations were used while the surface characteristics were different, so we can infer similar conclusions. From the aspect of carbon metabolism, this research focused on the effects of different surface modifications of PS NPs on the function of biofilms, which revealed that the total carbon metabolic activity of biofilms decreased significantly in the polystyrene-introduced environment, and there were distinct responses of the six biochemical categories of carbon source to these two types of PS NPs. These results suggested that different surface chemical properties of polystyrene lead to an influence on freshwater biofilms in varying degrees.

In this study, we found that the inhibited effects of PS NPs on biofilm metabolic activities were obviously dependent on the surface modification, likely due to the binding affinity between PS NPs and the cells [21]. Accordingly, the positively charged nanoplastics were found to show a higher adsorption to algae than negatively charged particles [31]. Also, it has been documented that negatively charged PS nanoparticles exhibited a lower binding affinity to the cell wall of Pseudokirchneriella subcapitata than non-functionalized and positively charged PS nanoparticles [21]. Therefore, more studies should be performed to investigate the potential impacts of PS NPs with various surface modification on microbial communities in aquatic environments.

### 4.2. Environmental Implications

Generally speaking, the results drawn from the exposure experiments in the long term and with a low concentration of plastic particles could be more useful to assess the effects in the freshwater system. Although there is limited information about the environmental concentrations of PS NPs in freshwater, the applied concentration of PS NPs in this study are likely to be higher [32], indicating that the extrapolation of our results requires caution. Guo song et al. found that with the increase of NP concentrations, the total carbon metabolic functions (expressed as AWCD) remained unchanged, while the utilization capacity of some specific carbon sources (e.g., esters) changed. At 10 mg/L NPs, microbial functional diversity (Shannon Wiener diversity index, Simpson diversity index and Shannon evenness index) decreased significantly [16]. 

In this study, 10 mg/L suspension solutions were prepared for an exposure experiment in order to clearly show the changes of biofilm functional characteristics. However, due to the increasing release of nanoplastics into freshwater, detailed and comprehensive studies should be conducted to investigate their ecotoxicological effects. From the present study, the toxic effects of PS beads of different surface modifications on freshwater biofilms after 21 days of exposure was explored and the related mechanisms were discussed. Distinct responses of biofilm carbon metabolism to nanoplastics with different surface modifications were observed. Previous studies have shown that NPs can damage membrane structures and affect molecular diffusion and even gene expression revealed by GeoChip 5.0 [33]. More studies that aim to investigate the effects of NPs on the community structure and microbial functions of biofilms could provide a better understanding about the effects of NPs in freshwater environments.

## 5. Conclusions

While research on the effect of nano-plastics to microbial communities has drawn increasing interests, knowledge of the responses of microbial functions related to the biogeochemical cycles of biofilms is still limited. Herein, the toxic effects of PS NPs on biofilms were observed to be strongly dependent on their surface modification. Furthermore, clear differences in metabolic characteristics were elucidated for six biochemical categories of 31 carbon sources, indicating that the consumption preference changed in the microbial community and that nano-plastics with different surface modifications have different degrees of effects on the metabolic capacity of specific carbon sources. These results highlight the significant risks posed by PS-NPs of different surface chemical properties on the ecosystem functioning of biofilms, which should be further studied.

## Figures and Tables

**Table 1:** Particle size distribution (*n* = 3, mean particle diameter ± SD) and zeta potential of PS NPs.

Polystyrene Type	Particle Size Distribution (nm)		Zeta Potential (mV)	
	Milli-Q Water (pH 6.9 ± 0.1)	Experimental Solution	Milli-Q Water	Experimental Solution
non-functionalized PS	122 ± 34	563 ± 124 *	−38.4 ± 3.3	−19.7 ± 4.5 *
PS-COOH	131 ± 16	681 ± 152 *	−40.3 ± 4.8	−19.6 ± 3.2 *

* an asterisk demonstrates significant differences of mean diameter and zeta potential for the PS NPs in the experimental solution compared with those in Milli-Q water (*p* < 0.05).

## Data Availability

Data will be availability when requested.

## Supplementary Materials

The following supporting information can be downloaded at: https://www.mdpi.com/article/10.3390/ijerph19159148/s1.
