# Supplementary material for: Distinct Responses of Biofilm Carbon Metabolism to Nanoplastics with Different Surface Modifications
Source: Int J Environ Res Public Health. 2022 Jul 27;19(15):9148. doi: 10.3390/ijerph19159148 (PMC9332795; doi:10.3390/ijerph19159148)
Supplement: Supplementary file 1 [file ijerph-19-09148-s001.zip › ijerph-1762459-supplementary.pdf]

Supporting Information for

Distinct responses of biofilm carbon metabolism to nanoplastics with different surface modifications

Yang Liu[1,2], Yuwei Li[3], Chunmei Tao[4], Junjie Zhao*[5], Hongmei Zhang*[6], Lingzhan Miao[3], Yong Pang[1], Jun Hou[3]

[1] College of Hydrology and Water Resources, Hohai University, Nanjing 210098, Jiangsu, China
[2] Jiangsu Environmental Engineering Technology Co., Ltd., Nanjing 210036, China;
[3] Key Laboratory of Integrated Regulation and Resources Development on Shallow Lakes, Ministry of Education, College of Environment, Hohai University, Nanjing, People's Republic of China, 210098
4 Lianyungang Water Conservancy Bureau, Lianyungang, 222006, China,
5 Key Laboratory of Waterway Traffic Environmental Protection Technology, Tianjin Research Institute For Water Transport Engineering, M.O.T, Tianjin, People's Republic of China, 300456
Shannan Ecological environment Monitoring Center, Tibet, China, 856100,

* Correspondence:
Junjie Zhao，E-mail: ppurelife@163.com;
Hongmei Zhang, E-mail: 504175589@qq.com;

Table S1. WC Medium Recipe

Parameter	Concentration
$NaNO_3$	1 mM
$CaCl_2 \cdot 2H_2O$	0.25 mM
$MgSO_4 \cdot 7H_2O$	0.15 mM
$NaHCO_3$	0.15 mM
$Na_2SiO_3 \cdot 9H_2O$	0.1 mM
K_2HPO_4	0.05 mM
H_3BO_3	0.39 mM
WC Trace Elements Solution	1 mL/L
Vitamin B_{12}	1 mL/L
Thiamine Vitamin Solution	1 mL/L
Biotin Vitamin solution	1 mL/L

Text

BIOLOG ECO microplates (Hayward, CA, USA) are a well-known effective detector to evaluate selection and utilization ability of microbial carbon source. There are 96 wells in the microplates consisting of three parallel systems for repeated tests, which involve a blank well and 31 different sole carbon source wells loaded with the same content of tetrazole violet dye. To be specific, the 31 carbon sources can be classified into5 biochemical categories: carbohydrates, polymers, carboxylic acids, amino acids and amines/amides. When different species oxidize carbon sources, electrons transferred will be captured by tetrazole purple fuel and thus turning purple. The color change can also be qualified using absorbance at 590 nm (OD 590). Average well color development (AWCD) is used to assess the ability of the microbes to metabolize the carbon sources.

Calculation of metabolic functional diversity indices

The metabolic diversity of communities can be reflected from the number of carbon sources and their utilization capacity on certain specific plates. A calculation based on the functional diversity indices of the BIOLOG ECO microplates can be applied to determine the metabolic potential of microbial communities (Ge et al., 2018; Miao et al., 2019b). There are three metabolic functional diversity indices listed below:

(1) The Shannon–Wiener diversity index (H'):

$$H = -\sum P_i \ln P_i \qquad (1)$$

$$P_i = (C_i - R)/\sum(C_i - R) \qquad (2)$$

where, *Pi* is the relative ratio of the absorbance value in the *ith* (1–31) well to the total

absorbance values of all wells.
(2) The Simpson diversity index (D):

$$D = 1 - \sum P_i^2 \quad (3)$$

(3) The Shannon evenness index (E):

$$E = H' lnS \quad (4)$$

where, *S* is the total number of utilized carbon sources (31 carbon sources).
The three metabolic functional diversity indices mentioned above can reflect the metabolic diversity of microbial communities, and are widely adopted to interpret the combined effects of richness and evenness in ecological research.

1. Ge, Z., Du, H., Gao, Y., Qiu, W., 2018. Analysis on Metabolic Functions of Stored Rice Microbial Communities by BIOLOG ECO Microplates. Front. Microbiol. 24. Miao, L., Wang, P., Hou, J., Yao, Y., Liu, Z., Liu, S., et al., 2019b. Distinct community structure and microbial functions of biofilms colonizing microplastics. Sci. Total Environ. 650, 2395–2402.
2. Miao, L., Wang, P., Hou, J., Yao, Y., Liu, Z., Liu, S., et al., 2019b. Distinct community structure and microbial functions of biofilms colonizing microplastics. Sci. Total Environ. 650, 2395 - 2402.

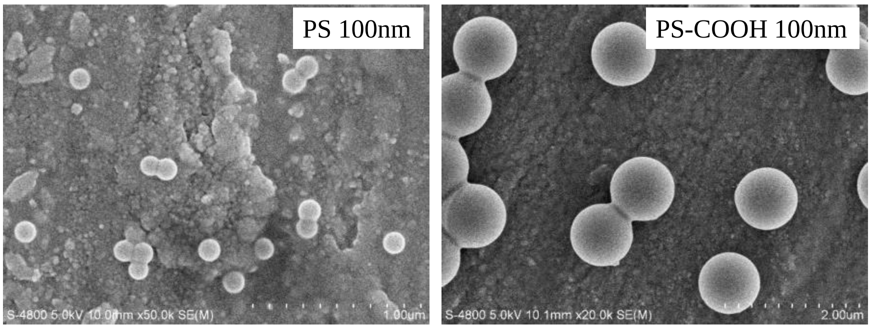

Figure S1. The SEM images of the applied PS NPs (non-functionalized PS and carboxyl PS) in this study.
